# Doctors’ experiences providing sexual and reproductive health care at Catholic Hospitals in the conflict-affected North-West region of Cameroon: a qualitative study

**DOI:** 10.1186/s12978-022-01430-w

**Published:** 2022-05-28

**Authors:** Adama Awasom-Fru, Maturin Désiré Sop Sop, Elin Charlotte Larsson, Sibylle Herzig van Wees

**Affiliations:** 1 SWEDESD - Sustainability Learning and Research Centre at the Department of Women’s and Children’s Health, Uppsala University, Uppsala, Sweden; 2 Department of Geography, University of Bamenda, Bamenda, Cameroon; 3 Department of Global Public Health, Karolinska Institutet, Stockholm, Sweden; 4 Department of Women and Childrens Health, Karolinska Institutet, Stockholm, Sweden

**Keywords:** Sexual and reproductive health, Doctor’s experiences, Catholic hospitals, Cameroon

## Abstract

**Background:**

Sexual and reproductive health (SRH) care services are essential to improving the lives of women and achieving the Sustainable Development Goals. In Cameroon, the Catholic Church is one the largest non-governmental suppliers of health care, but its role in providing SRH care is restricted by religious norms.

**Methods:**

This study explored doctors’ experiences and perceptions of providing SRH care at Catholic hospitals in a conflict-affected area in Cameroon by using 10 in-depth interviews with doctors from three Catholic hospitals in the North-West region. Qualitative coding was done with NVivo, and data were analysed using thematic analysis.

**Results:**

Three themes and seven categories were identified. The respondents described strict rules and a broad range of challenges to providing comprehensive sexual and reproductive health care services. Nonetheless, there is evidence of doctors overcoming obstacles to providing SRH care despite the religious and political climate. However, whilst attempting to overcome challenges, participants described numerous examples of poor SRH care and health outcomes.

**Conclusion:**

The study highlights the importance of understanding the intersect between religion and women’s health, particularly in improving access to SRH for vulnerable populations in conflict-affected areas. It further provides insight into doctors’ motivations in practicing medicine and how doctors cope and make efforts to provide care and minimize harm.

## Plain English Summary

The main aim of this study was to explore doctors’ experiences and perceptions of providing sexual and reproductive health care services at Catholic hospitals in a conflict affected area in Cameroon. In Cameroon, sexual, and reproductive health services are lacking, thereby contributing to a very high maternal mortality rate of about 456/100000 live births, far from the Sustainable Development Goal Agenda 2030. The Catholic Church remains one of the largest suppliers of health care services in Cameroon and receives huge funding from donors, but their role in providing sexual and reproductive health care is restricted by religious norms. This study therefore uses in-depth interviews with doctors from Catholic hospitals in the North-West region to explore their experiences in providing sexual and reproductive health services in a conflict region. Qualitative coding was done with NVivo, and data analysed using thematic analysis. The respondents described strict rules and a broad range of challenges to providing comprehensive sexual and reproductive health care services. Nonetheless, there is evidence of doctors overcoming obstacles to providing sexual and reproductive health (SRH) care despite the religious and political climate. However, while attempting to overcome challenges, participants describe numerous examples of poor SRH care and poor health outcomes. The study highlights the importance of understanding the intersect between religion and women’s health, particularly in improving access to SRH for vulnerable populations in conflict affected areas. It further provides insight into doctors’ motivations for practicing medicine, and how doctors cope and make efforts to provide care and minimize harm.

## Background

Sexual and reproductive health and rights (SRHR) are essential to promoting gender equality and ensuring good health for all and are integral to achieving the Sustainable Development Goals (SDGs), especially goals 3, 4 and 5 [1]. Accessing top quality SRHR services is essential to improving women’s lives worldwide, particularly in Sub-Saharan Africa, where a high burden of disease and mortality is associated with limited access to SRHR [2]. Reproductive health influences a woman’s quality of life, and lack of access to SRHR services can lead to unintended pregnancies, unsafe abortions, sexually transmitted infections, gender-based violence, disability and maternal death [3].

Faith-based health providers are organisations that deliver health services to their populations, but these services are underlined by principles and morals based on religious beliefs [4]. Faith-based health providers have a long-standing history of providing health care to populations in many countries in Sub-Saharan Africa, where reports have estimated that these providers contribute about 30‒70% of all health care [5]. Faith-based health providers have been able to reach extended communities, assisting people most in need by complementing services provided by the public health sector [6]. They have also been involved in sexual and reproductive health care services, especially providing family planning and HIV/AIDS services in Sub-Saharan Africa. For example, the Global Fund to Fight Aids recognized the importance of faith-based actors as partners and has channelled almost 2 billion dollars through these organisations to strengthen programs aimed at addressing AIDS, tuberculosis and malaria [7]. Some reports have claimed that about 25% of all HIV/AIDS-related services worldwide are provided by the Catholic Church [8].

The Catholic Church is one of the largest non-governmental providers of health services in the world [6]. However, all institutions under the umbrella of the Catholic Church must serve the population in accordance with the norms and values of the church [9]. These directives are stringent and allow little flexibility regarding SRHR [10, 11]. The Catholic Church, for example, forbids the use of all forms of modern contraception, including sterilization and the use of condoms [12], and further forbids abortions when directly intended to end a pregnancy after conception [13]. Moreover, Catholic health care services are prohibited from providing access to emergency contraceptives [14]. Instead, the Catholic Church promotes abstinence for the unmarried and encourages fidelity among married couples as the only morally acceptable and most effective way to prevent sexually transmitted infections [15]. The Catholic Church does not support comprehensive sexual education and argues that it is the right of the parents to educate their children on matters of sexuality, with emphasis on teachings that obey the moral obligations of its doctrine [10].

The Ministry of Public Health governs the Cameroonian health system. Health service is provided by public, private and traditional health care services [16]. About 40% of health care delivery provided by the private sector is composed of faith-based actors, NGOs and private institutions [17]. The Catholic Church plays a pivotal role in health service provision in Cameroon and provides about a third of all faith-based health care in the country [18]. Faith-based health providers hold a good reputation in Cameroonian communities, particularly for their quality of care and compassionate service [19]. Despite strict rules and norms surrounding SRHR, in the recent decade in Cameroon, faith-based health providers, including Catholic health providers, have been engaged by donors in several sexual and reproductive health programmes [20]. For example, Catholic health providers have been included in performance-based financing initiatives funded by the World Bank to increase access to sexual and reproductive health care, HIV/AIDS programmes as well as midwifery training [21]. In spite of this increased engagement of faith-based actors by donors, SRHR indicators in Cameroon continue to be poor, with an unmet need for family planning at 23% and high maternal mortality of 467/100,000 live births, far from the goal set by the SDG [23]. There is also some evidence to suggest that SRHR efforts have not been successful among Catholic health providers in Cameroon [22]. The North-West region of Cameroon, where this study took place, has been severely affected by conflict since 2016 [24]. This conflict has further hampered access to health care services, including SRHR, as there has been a growing need to provide health care to the population [25]. Given these poor SRHR outcomes, the high unmet need and the continued programmatic support of Catholic hospitals, it remains important to understand the delivery of sexual and reproductive health services at Catholic hospitals, particularly in conflict-affected areas, which have additional limited access to health care.

## Research methods

### Conceptual framing

The conceptual framework that guided this study is situated at the interface between gender and religion, particularly Catholicism, and health care provision in fragile health systems. The research explores and builds on research on tensions between Catholicism and sexual and reproductive health and posits these in a context where the health system is fragile due to conflict [11]. Civil conflict has been shown to directly affect the health outcome of a population, leading to poor health outcomes, particularly in women. In regions with already fragile health care structures, conflict further exposes these weaknesses and leaves the population stranded. A study on post-war effects of conflict showed that long-term death and disability due to direct and indirect causes of civil war disproportionately affect women and children [26]. It draws on literature and builds on the idea that faith-based health care providers can reach communities in the context of conflict because they appear more flexible and manage to withstand the stress of the crisis [26–29]. This conceptual framework guided the following aim: to explore the experiences of doctors providing sexual and reproductive health care at Catholic hospitals in a conflict-affected region.

### Study design

This is a qualitative study using in-depth interviews to explore doctors’ experiences in delivering sexual and reproductive health care at Catholic hospitals. The study was reported following the Consolidated Criteria for Reporting Qualitative Research (COREQ) [30].

### Study setting

Cameroon is a lower-middle-income country located in Central Africa and has a population of about 26,545,863 inhabitants [31]. This bilingual (English-French) country comprises 10 administrative regions divided into 189 health districts [32]. The English-speaking part constitutes two of the 10 regions and makes up about 20% of the country’s population [33]. The population of Cameroon is 38.4% Catholic, 26.4% protestant, 20.9% Muslim and about 14% other beliefs [33]. The main health financing sources are the government, public enterprises, foreign aid donors, private enterprises, households, religious missions and non-governmental organisations (NGOs) [34].

This study was conducted in the North-West region of Cameroon, one of two anglophone regions. The anglophone regions have been undergoing what is controversially known as the “Anglophone Crisis”, characterised by civil protest, strikes, the evolution of armed groups and militarization of the region to date [24]. Human Rights Watch reports indicate that hundreds of thousands of civilians have been displaced and/or killed, including the deaths of many armed separatists and military personnel since the beginning of the crisis [35]. The crisis has caused the destruction of many health care structures in an already fragile health care system, leaving the population in the North-West region of about 2 million inhabitants [36] vulnerable to disease and death [25]. There are about 20 Catholic health providers in the North-West Region of Cameroon, of which only a few have remained functional since the onset of the crisis. These services have primary, secondary and tertiary health structures in the region. Three Catholic hospitals, which were still functional, were purposefully selected for this study based on their offering general consultation services, including sexual and reproductive health care services such as maternal and childcare services to the population. The hospital names are purposively omitted to protect the anonymity of the research participants. Cameroon was selected as a case study because it was accessible to the research team even though it is a conflict-affected area.

### Study population and sampling

Purposive sampling was used to select 10 medical doctors working at three Catholic health care facilities. Selection criteria were general practitioners who have worked at a Catholic hospital for at least one year. General practitioners were selected because they have first-hand exposure to all patients who present at the hospital, including women seeking sexual and reproductive health care services. Specialists were excluded because the range of patients they see is specific and would not fit the scope of this paper. The participants included three female and seven male doctors available for the study; their working experience in Catholic hospitals ranged from two to seven years. We reached the maximum number of participants at these hospitals. Selecting additional sites was not possible due to security reasons.

### Data collection

All interviews were conducted in English by AAF, who used an interview guide (Annex 1) within a 6-week period. Some interviews were conducted over Zoom, whilst others were done at the doctors’ offices. Interviews lasted approximately 40 min and were recorded using a mobile phone and computer. The interviews were transcribed. Information was stored in the researcher’s laptop and safely kept using a password known only by the principal investigator to ensure the confidentiality of sensitive information.

### Data analysis

Data were analysed using the principles of thematic analysis by Braun and Clark [37]. The analysis began by transcribing the recorded data within four weeks of the in-depth interviews. The recorded data was listened to twice to ensure information was not lost and underlying meaning was represented in the transcription. The data was re-read, making notes of explanations.

Double open coding was done by AAF and SHvW with the assistance of NVivo version 12.0 [38]. Initial coding involved analysing the meaning of the text, including all data that were potentially relevant to the research aim, into different or similar codes. The next stage of coding included recoding and rechecking original codes. After this, similar codes were grouped into subcategories, categories and themes. Every subcategory consists of at least five quotes to illustrate the subcategory. In other words, a subcategory was only created if we found repetition in the data. Later, the researchers re-examined and re-evaluated the themes and categories to ensure there was no overlapping of ideas and the interpretation within and between the themes were coherent. The review of themes and categories by the team led to some changes by looking for the latent meaning of the data and resulted in merging, deleting and renaming some subcategories and themes. After repeating this process a few times and ensuring that each theme had a distinct focus, was not repetitive and answered the research question, a final version of three themes was developed.

### Ethical consideration

Ethical approval (2021/292H/uba/IRB) was sought from the ethical review board of the University of Bamenda, Cameroon, to conduct this study (Annex 1). Permission was also sought from each hospital to allow the researcher to conduct interviews with their staff. All participants were informed of their right to participate or not in the research project and their right to withdraw at any time during the duration of the project. All interviewees signed a written consent form and agreed to audio-recording.

### Reflexivity

During the research process, researchers must be aware of their role in conducting the research and analysis and how this might influence the outcome of the study. AAF has previously worked as a doctor at a Catholic hospital; consequently, the participants felt at ease and were open to discussing sensitive issues with her. AAF also tried to ensure that the information collected accurately represented the participants’ ideas by asking many follow-up questions for clarity. Additionally, AAF made a conscious effort not to express her opinion during the interview and analysis process. Avoidance of personal bias was possible through regular reflections in a research diary and continuous discussion of progress and findings with the research team.

## Results

The qualitative data analysis resulted in three major themes presented in Table 1. Several quotes support each sub-category; the most illustrative quotes were selected to show the findings of the sub-categories and categories.

**Table 1:** Summary of themes, categories and sub-categories

Theme	Category	Sub-category
Navigating rules and dilemmas when providing SRHR	Rules	Institutional rules: SRHR at Catholic hospital Atmosphere of fear Responsibility towards the patient and community (people want SRHR services) Responsibility towards the administration Personal faith
	Dilemmas	
	Negotiating with multiple partners to find solutions	Administration agreeing to some procedures Partnership with other hospitals or organisations Referring patients Advising patients Providing SRHR care in secret
Overcoming obstacles to providing SRHR care	Doctors put patients first	
Poor SRHR and outcomes	Unfortunate effects of conflict Unsatisfactory SRHR care Tragedies and abandonment	Patient overload Poor post-rape care Missed opportunities Crisis effects

### Navigating rules and dilemmas when providing SRHR

When working at Catholic hospitals, doctors are expected to abide by the rules of the Church. These rules include a ban on advising, prescribing or performing any form of modern family planning within the hospital and a ban on the prescription of emergency contraceptives in the event of rape or incest. Abortions, although not only limited to Catholic hospitals in Cameroon, are also strictly prohibited, even in the case of medical necessity. Instead, if patients request information about family planning, they receive counselling on the natural family planning method from a reverend sister. To summarise in the words of one of the participants.

*…we see patients who come in for family planning or just counselling on the reproductive health…you have a limited number of things that you can say to that patient, as a doctor working in a Catholic hospital. For example, like the Catholic Church pushes only for the natural method of contraception that is a woman knows her safe and unsafe days. (Interview 2)*

Some doctors learned these rules prior to their practice, whilst others were expected to learn about them during their practice. The strict rules and regulations affect the working environment, which the interviewees describe as an environment of fear from the religious hierarchy.

…I know many health personnel who shy away from interaction with the hierarchy. … There is this fear. You're working with fear…. (Interview 1)

This environment of fear influences practice regarding SRHR. Doctors must navigate rules from the administration, best practices in medicine, what is best for their patients, as well as their own beliefs, which constantly leads to dilemmas.

*… it's very difficult to observe everything as a Catholic or everything as a medical doctor. So, you have to join between the two and know what is good for the patient. (Interview 3)*

The doctors described receiving constant demands and desperate requests from patients who need SRHR care and are unaware that these services are not provided at Catholic hospitals. These demands primarily concerned emergency contraceptives in young adults and sterilization for women with multiple children. For example, where a woman with multiple previous caesarean sections needed sterilization, the doctor felt that rejecting assistance could lead to subsequent high-risk pregnancies and even death. These situations were troubling for many doctors interviewed in this study, especially those who were Catholics, when trying to find a balance between their faith and moral duties to the patient.

*It makes it difficult, you know, when you see someone who is in need, you see patients, and because of some rules, you cannot cater to the patient. There is this saying that the patient is the king. So, do you tell the king, ‘Sorry, I cannot serve you?’ (Interview 4)*

### Overcoming obstacles to providing SRHR care

Nevertheless, despite the regulations regarding SRHR provision at Catholic hospitals, doctors find ways to navigate these challenges and provide services to the best of their abilities. Three ways of doing so emerged from the data analysis.

Firstly, doctors opt to refer patients to government hospitals or other hospitals that offer SRHR services they cannot. Some Catholic hospitals were against referrals but had no way to monitor referrals by doctors since these were done during consultations, whilst other Catholic hospitals considered referral an appropriate alternative.

*The truth is that we are there for the patient. And if the hospital has a policy that they don't permit this contraceptive to be offered here. But you see that that patient needs it. So, I advise the patient that this is where you can get it, and this is where you can get a better management. (Interview 2)*

Doctors would also refer victims of sexual violence to non-governmental or international organisations that provide emergency contraceptives. In the words of one of the interviewees:

*The Church says you should not give the patient (emergency) contraception, but it doesn't say whether you should tell them about the availability of contraception. So, there's this grey area that you can manoeuvre. I can tell her that we cannot give, but you can go somewhere … it boils down to their choice whether they want to have it or not. (Interview 8)*

Secondly, doctors have managed to cooperate with the Catholic hospital administration to assist patients. If the doctors are able to demonstrate a potential direct risk to a woman’s life if an intervention is not done, some administrations agree to allow some interventions.

*I've had a patient that has had an ectopic pregnancy on an intrauterine device. For the case of ectopic pregnancy, she was 46 years, and she wanted to stop having children. …So, in that case, I had to operate. And because of that, I advised the institution that it was better for us to do the tubal ligation [female sterilization]. After several conversations, they finally accepted. (Interview 5)*

However, such approval was highly dependent on the administration involved, as some of them denied such requests.

A third way to overcome challenges was repeatedly described as how doctors put patients first and provided SRHR without the involvement of their institutions. In other words, they would break the rules to help a patient. In some instances, the doctors and their colleagues discussed ways to assist patients. This help came in the form of performing procedures within the hospital milieu in secrecy, advising patients on all the options available for any SRHR services and/or referring patients to hospitals where doctors knew the patients could receive appropriate care.

*You know, we have our own code. In as much as you are in a held institution, we have our small community as doctors where we have situations we first of all discuss among ourselves as doctors. (Interview 3)*

Moreover, doctors mentioned that they mostly avoid conflict surrounding this topic and get on with their role as doctors.

You have to maybe act like you agree with the hierarchy, but deep down, you're the person in contact with the patient. (Interview 10)

### Poor SRHR care and outcomes

Even though doctors do their best to address SRHR needs at Catholic hospitals, they describe a high unmet need, suboptimal SRHR care and dangerous practices in the region affected by conflict.

Doctors describe a remarkably high unmet need for SRHR services which has worsened during the crisis in the North-West region, firstly because of less available functional hospitals, overcrowding of existing hospitals, and impoverishment of people due to the conflict who are desperate to receive health care.

*…the crisis has made everything worse. The people here don’t have any money to pay for bills. It’s not even safe for them to go to the farm to earn a living. Many of them in the other villages ran away, including myself. All the hospital staff left. I heard my previous hospital is now a hiding spot for the fighters. It’s very complicated. (Interview 2)*

With increased demand for and reduced access to health care, doctors describe several missed opportunities where women could prevent more unwanted pregnancies, such as immediately after delivery of a baby or post-rape. The doctors believe that the availability of postnatal counselling for family planning could prevent the observed early repeated pregnancies.

Women are already pregnant again when they come to vaccinate their babies at eight weeks old. But now, you cannot do anything about it in the hospital. (Interview 9)

Moreover, even in cases where referrals could be seen as an option, doctors are concerned about missed opportunities for care due to the referrals they sometimes make for modern family planning, especially in times of conflict. The distance to the other hospitals, long waiting times and limited availability of contraceptives begs the question of whether patients get access to these services despite referrals. This limited access to contraceptives becomes even more frustrating when it comes to appropriate post-rape care, which is time-dependent.

*… after rape, there's a timeframe in which you need to take the medication, so when they come to us, by the time we've done all the tests, talked to them, made them feel better, and referred them to pharmacy, it takes a very long time. Sometimes they don’t even want to leave when they come to the hospital; they spend hours and hours crying, and we might have to find a family member who will need to go and buy the medications. If it [emergency contraception] was readily available, something that the moment they come, and they've been raped, that's the first they should take because it depends on the hours, right..Yeah, I wish I really used to feel like we could have done more. (Interview 7)*

Doctors further describe challenges around providing SRHR care in secrecy. This poses a problem, especially regarding recording the patient medical history. In extreme cases, doctors exaggerate or falsify medical records to justify reasons to provide some services such as sterilization. At other times, they carry out procedures and do not record them in the file to avoid getting caught by the administration.

*Yeah, they had like, for example, there are cases where because there are some reverend sisters [nuns in charge of the hospital] who, when they are present in the theatres, know exactly what we're doing. And the woman really, really wants bilateral tubal ligation [female sterilization]. So, we just discuss with our colleagues and discuss with a patient, and we'll be like, okay, you said you had three surgeries. Just say you have four surgeries; we would write that in your book so that the sisters can allow us to do this for you. (Interview 8)*

Doctors further describe challenges in managing the implications of improvising and bending the rules. For example, what if a patient changes their mind and decides to report their covert activities to the administration? Moreover, doctors have to communicate with each other to ensure continuity of care, especially since medical records cannot include certain information.

*… the rest of us have to know what was done because we sometimes follow up the same patient. We need to have the same language in front of the sisters, right, so we don’t tell one sister something, and then other doctors say actually, this patient didn’t go through this procedure, and you get in trouble. (Interview 6)*

The doctors explain that the consequences of not being able to provide SRHR freely are not always optimal. It can lead to anger and frustrations during work, especially if a patient loses their life over a preventable cause.

*There was an incident that happened with a girl who was pregnant. She was about 19 years old and wanted to do a pregnancy test. Unfortunately for her, there was a reverend sister there; she did a test. The reverend sister refused to tell her the results and told her to call her parents. So, this girl refused to make the call, got desperate and left … I don't know what she did… this lady came back to the hospital about a week later and had developed sepsis... she had an unsafe abortion… And we couldn't save her life. (Interview 10)*

## Discussion

This study focuses on the experience and perceptions of doctors in providing sexual and reproductive health care in Catholic hospitals in conflict-affected areas in Cameroon. The findings echo previous literature pointing to the challenges in the provision of SRHR at faith-based health providers [39, 40]. For example, a reproductive health programme in Burkina Faso examined the effect of faith-based actor engagement on gender inequality and health; it was found that the faith-based actors did not provide condoms [41]. Moreover, in the context of the studied community, it is socially acceptable for men to have extra-marital affairs, but not women. The faith-based actors do not account for these gender imbalances, which leave women more vulnerable to unwanted pregnancies and sexually transmitted infections, including HIV/AIDS [41].

This study further shows that doctors disagree with strict hospital policies about SRHR and continually face dilemmas where they must balance the needs of the patients, regulations of the hospital and personal beliefs. Just as in other studies, health care workers in faith-based hospitals do not always agree with hospital policy and often face difficult ethical decisions in their patient management [40, 42, 43]. This sometimes causes them distress, especially when they feel the patient’s outcome is a direct consequence of their actions [44, 45].

One of the core findings of this study is that doctors find ways to overcome the barriers to providing sexual and reproductive health services at Catholic hospitals. Whilst this is a promising finding and arguably increases women’s access to the services they need, there are many problems with the clandestine provision of sexual and reproductive health care. This is problematic in a setting where resources are scarce, and it takes time to find ways to provide the services without involving the administration [16]. It also complicates issues such as health record management, which could pose problems if post-operative complications emerge. Moreover, patient referral may be the solution doctors highlight, but given the transportation challenges in the North-West region and the insecurity linked to moving around, it is not likely that women are able to seek public services that offer the sexual health services they require [25, 46].

Consequently, the finding that doctors continuously observed poor sexual and reproductive health outcomes, including the inability to provide emergency contraception on time, the inability to advise young women on family planning, witnessing backstreet abortions and the consequences thereof, is not surprising‒and similar to findings in Cameroon [47, 48].

The results from this study highlight that there is a treatment gap for SRHR needs and that Catholic values on SRHR undermines good quality SRHR care. This finding is important given that donors invest substantially in faith-based health providers in Cameroon [20, 22]. It is also a critical finding due to the growing treatment gap resulting from enduring conflict in the region. Whilst we do not argue for discontinuation of donor investment, we argue for more dialogue on this topic and exploration of solutions, particularly in times of conflict where resources are scarce and SRHR needs are especially high. For example, referrals appear to be an acceptable solution (although not always) for Catholic hospitals. However, these referrals are expensive, not systematic and without follow-up. Systematic and specific collaborations between Catholic and public hospitals or between Catholic hospitals and non-governmental actors that provide services such as emergency rape care should be encouraged. We also suggest that doctors receive guidance and support on facing dilemmas at the interface between religion and medicine as described in this study as part of their medical education.

A further important finding is that despite the fact that the conflict requires adaptation and responses to growing needs for women’s health, the results in this study show that adaptation does not happen, and Catholic norms and values prevail. This is contrary to previous literature in which faith-based health care providers have shown to be more resilient in providing health care, especially during conflict, and complement public sector activities [27, 28]. A study conducted in Ghana, Malawi, DRC and Sudan, for example, showed that faith-based health care providers play a significant role in strengthening the health care system and are able to withstand the stress of crises [38, 40]. The results from our study show that although Catholic hospitals are important in the context of a conflict, they do not address specific sexual and reproductive health needs of vulnerable women.

### Limitation

The limitation of this study is that we were only able to talk to 10 participants. We planned to talk to about 15 participants. However, the conflict in the North-West region made it difficult to collect data. First, communication was limited due to electrical power cuts. Also, doctors’ time for research was limited due to the high workload at understaffed facilities, so it was considered unethical to continue to ask them for their time under challenging circumstances. Despite this limitation, we believe that saturation started to be reached at interview number eight; in other words, we saw the repetition of findings in the interviews after only eight interviews.

## Conclusion

This study shows that doctors try to overcome barriers to providing sexual and reproductive health care at Catholic hospitals. However, doctors remain frustrated with treatment dilemmas, poor health outcomes, tragedies and missed opportunities for improved sexual and reproductive health care. Women further lose out to urgently needed sexual and reproductive health services. The findings from this study show that innovative solutions need to be evaluated to address both doctors’ difficult positions and missed opportunities for much-needed sexual and reproductive health care, particularly in conflict-affected areas.

## Data Availability

The datasets generated and/or analyzed during the current study are not publicly available due sensitive nature of contents and confidentiality but are available from the corresponding author on reasonable request.

## Abbreviations

SRH: Sexual and reproductive health
SRHR: Sexual and reproductive health rights
RH: Reproductive health
SDG: Sustainable development goals
FBPs: Faith-Based Organizations
WHO: World Health Organization
SSA: Sub-Saharan Africa
HIV/AIDS: Human immune deficiency virus/acquired immune deficiency syndrome
